# A New Exon Derived from a Mammalian Apparent LTR Retrotransposon of the *SUPT16H* Gene

**DOI:** 10.1155/2013/387594

**Published:** 2013-03-10

**Authors:** Min-In Bae, Yun-Ji Kim, Ja-Rang Lee, Yi-Deun Jung, Heui-Soo Kim

**Affiliations:** ^1^Department of Biological Sciences, College of Natural Sciences, Pusan National University, Busan 609-735, Republic of Korea; ^2^Department of Nanobiomedical Science & WCU Research Center, Dankook University, Cheonan 330-714, Republic of Korea

## Abstract

The *SUPT16H* gene known as FACTP140 is required for the transcription of other genes. For transcription, genes need to be complexed with accessory factors, including transcription factors and RNA polymerase II. One such factor, FACT, interacts with histones H2A/H2B for nucleosome disassembly and transcription elongation. The *SUPT16H* gene has a transcript and many expressed sequence tags (ESTs). We were especially interested in an MaLR-derived transcript (EST, BX333035) that included a new exon introduced by a transposable element, a mammalian apparent LTR retrotransposon (MaLR). The MaLR was detected ranging from humans to galagos, indicating the MaLR in the *SUPT16H* gene is integrated into the primate ancestor genome. A new exon was created by alternative donor site provided by the MaLR. The original transcript and the MaLR-derived transcript were expressed in various human, rhesus monkey, and other primate tissues. Additionally, we identified a new alternative transcript that included the MaLR, but there was no significant difference in the expression of the original transcript and the MaLR-derived transcript. Interestingly, the new alternative transcript and the MaLR-derived transcript had the MaLR sequence in the new exon, but they had different structures by adopting different 3′ splice sites. From this study, we verified transposable elements that contributed to transcriptome diversity.

## 1. Introduction

Transposable elements (TEs) dispersed in the mammalian genome are on the rise, as completion of the whole human genome sequence. Contrary to our expectations, TEs, thought to be “junk DNA,” were estimated to account for approximately 45% of the human genome (International Human Genome Sequencing Consortium, 2001). TEs can be categorized by various features such as the capacity to mobilize themselves, DNA/RNA intermediation, and the existence of a long terminal repeat (LTR). Endogenous retroviruses (ERVs) and long interspersed elements (LINEs) have gene-encoding enzymatic machinery that could mobilize to other genomic regions as autonomous elements. However, nonautonomous elements, especially Alu elements, which are classified as short interspersed elements (SINE) and SINE-variable number tandem repeat-Alu-like sequences (SVA), mobilize using the reverse transcriptase of the LINE. All of the TEs described above are integrated into the host genome by their RNAs, but the integration of DNA transposons is mediated by DNA. These groups can be restructured by the existence of LTRs on both ends. ERVs and LTR retrotransposons have LTRs, but SINEs and LINEs are non-LTR elements [1–3].

One group of retrotransposon-like elements, mammalian apparent LTR retrotransposons (MaLRs), accounts for approximately 3.65% and 4.82%, including solitary LTRs, of the human and mouse genome, respectively [4]. The structure of MaLRs is similar to that of ERVs with LTRs. However, MaLRs can be distinguished from other retroviruses by some differences. Typical ERVs consist of an internal region, gag, pol, and env with LTRs on both sides. LTRs are very important for integration and transcription because they function as a promoter element, transcription start site, and polyadenylation signal and site [5]. Compared to ERVs, MaLRs have short internal sequences and LTRs at the 5′ and 3′ ends, but they do not have a transcription initiation site, reverse transcriptase, and primer binding site [6]. Recent studies have shown that TEs contributed to primate genome evolution through gene structure variation and regulation by integrating into an intron, gene-flanking region, and an exon [7, 8]. Additionally, TEs that integrate into introns could make alternative exons or cassette exons in a process termed “exonization” within human and mouse protein-coding genes [9]; in contrast, TEs can make new introns in a process called “intronization.” These events could help with the understanding of transcriptome and proteome complexities [9–11]. The exonization of the primate-specific Alu element is well established in the divergence of human and chimpanzee genomes as a major mechanism of exon creation [12, 13]. Lev-Maor et al. (2003) reported that the Alu consensus sequence could provide a splice site, leading to exonization [14]. In the case of LTRs, Piriyapongsa et al. (2007) reported that 256 of 1057 LTR retrotransposons related to human genes were observed in protein-coding regions, and 50 protein-coding exons of 45 genes were derived wholly from LTR retrotransposons. Furthermore, they analyzed alternative exons of the interleukin 22 receptor, the alpha 2 gene (*IL22RA2*), which was derived from MaLRs that provided splice sites, indicating the important roles of TEs in human evolution; this result coincided with those of previous studies [1, 5]. Importantly, these structural variations of genes are associated with gene regulation and genetic disease [15].

Here, we identified a transcript that included an MaLR in the *SUPT16H *gene. The *SUPT16H* gene, also known as FACTP140, is a component of the facilitates chromatin transcription (FACT) complex. In eukaryotic cells, the transcription of genes requires RNA polymerase II and transcription factors with altered chromatin structure [16]. In order to initiate transcription, DNA needs to be in an open structure that is moderated by the alteration of chromatin with accessory factors, facilitating access to the DNA. FACT specifically interacts with histones H2A/H2B to affect nucleosome disassembly and transcription elongation [17]. As described above, the *SUPT16H* gene has an original transcript consisting of 26 exons. However, the integration of an MaLR created a new exon that was expressed transcriptionally (BX333035). Furthermore, we found a new alternative transcript, including the MaLR-derived exon. We assumed the time of integration of the MaLR into the genome by PCR amplification. All of the primates assayed had the MaLR in the *SUPT16H* gene, which was a natural result of its conservation in the mammalian genome. Additionally, we compared the structure and expression of 2 transcripts, including MaLR. The expression patterns of the 2 transcripts in several tissues of humans, crab-eating monkey, marmoset, and squirrel monkey were ubiquitous with no significant tissue specificity or species specificity. Structurally, the 2 transcripts included the exon-derived MaLR, but they adopted different splice sites, leading to exons of different sizes. Taken together, we showed that the MaLR could regulate gene expression at the transcriptional level, that is, TEs could provide transcriptome diversity, resulting in the proteome's capacity for diversification, which potentially occupies an important role in the host genome.

## 2. Materials and Methods

### 2.1. Computational Analysis

To identify the sequences related to the MaLR, we scanned the human reference genome sequence, human ESTs, and human RefSeq mRNAs. Using the MaLR consensus sequence as a query, we screened nonredundant databases through BLAST version 2.2.26+ to identify the novel hybrid transcripts of the MaLR and *SUPT16H *[18]. Then, we aligned the transcripts of the *SUPT16H* gene to identify the precise splicing patterns. TEs included in the *SUPT16H* transcripts and their genomic loci were identified using the RepeatMasker program (http://www.repeatmasker.org/), referencing a library containing the consensus sequences from Repbase Update [19]. Using the EST and RefSeq mRNAs, we accurately reconstructed the *SUPT16H* gene structure. To analyze the sequences of the various *SUPT16H *transcripts, we aligned them using the ClustalW program [20].

### 2.2. Preparing for DNA and RNA

Using the TRIzol reagent (Invitrogen), we extracted the total RNA from the cerebrum, cerebellum, pituitary gland, heart, lung, spleen, liver, pancreas, kidney, and urinary bladder of the crab-eating monkey; the heart, lung, brain, stomach, liver, kidney, pancreas, colon, spleen, and small intestine of the marmoset; and the heart, esophagus, diencephalon, small intestine, lung, pancreas, cerebrum, colon, and stomach of the squirrel monkey. The total RNA from human tissues, including the adrenal gland, cerebellum, whole brain, heart, kidney, liver, lung, testis, trachea, bone marrow, fetal brain, fetal liver, placenta, prostate, salivary gland, skeletal muscle, spinal cord, thymus, thyroid, and uterus, were purchased from Clontech. We isolated the mRNA from the total RNA by using PolyATract mRNA Isolation Systems (Promega).

We used the genomic DNA of the following species: (1) hominoids: common chimpanzee (*Pan troglodytes*), gorilla (*Gorilla gorilla*), orangutan (*Pongo pygmaeus*), and gibbon (*Hylobates agilis*); (2) Old World monkeys: Japanese monkey (*Macaca fuscata*), rhesus macaque (*M. mulatta*), bonnet macaque (*M. radiata*), mandrill (*Mandrillus sphinx*), mangabey (*Cercocebus agilis*), African green monkey (*Chlorocebus aethiops*), colobus (*Colobus guereza*), and langur (*Trachypithecus cristatus*); (3) New World monkeys: night monkey (*Aotus trivirgatus*), squirrel monkey (*Saimiri sciureus*), and common marmoset (*Callithrix jacchus*); and (4) prosimian: ring-tailed lemur (*Lemur catta*) and a galago (*Otolemur crassicaudatus*). These genomic DNA were isolated from the heparinized blood samples according to a standard protocol [21]. After anticoagulant, the buffy coat was isolated carefully. And then, proteinase K and phenol equilibrated with Tris-Cl were treated for extraction. The viscous aqueous was transferred and ammonium acetate and ethanol were added to form a precipitate. Lastly, the DNA precipitate was washed, dried, and dissolved in TE (Tris and EDTA). Dr. Takenaka (Primate Research Institute, Kyoto University) kindly provided genomic DNA of primates after conducting this procedure. 

### 2.3. PCR and Reverse Transcription (RT)-PCR Amplification

Using the genomic DNA of humans and 17 primates, the MaLR in the *SUPT16H* gene was amplified to analyze the integration time in the primate genome. The primer pair included GS1 (5′-AATTGGCGGAAAGGAGAAG-3′) and GAS2 (5′-GAGGCAGTCATTCCAGCTCT-3′).

*SUPT16H* transcripts were analyzed by RT-PCR amplification. M-MLV reverse transcriptase (Promega) with an annealing temperature of 42°C and an RNase inhibitor (Promega) were used for the reverse transcriptions of the transcripts. The *SUPT16H* transcripts were amplified with a primer pair from GenBank (accession number NM001033560) consisting of S1 (5′-AATTGGCGGAAAGGAGAAG-3′) and AS1 (5′-GAGGCAGTCATTCCAGCTCT-3′). The *SUPT16H* transcripts related to the MaLR were amplified with a primer pair from GenBank (BX_333035) consisting of S2 (5′-TCCAGTTCTCTGTTCTGCCA-3′) and AS2 (5′-TGTGTGGAGCTTCAAGCAAG-3′). The PCR samples were subjected to an initial denaturation step of 4 min at 94°C, followed by 30 cycles of PCR at 40 s of denaturation at 95°C, 40 s of annealing at 55°C, and 90 s of extension at 72°C, followed by a final extension step of 7 min at 72°C. As a standard control, *GAPDH* was amplified with a primer pair consisting of GPH-S (5′-GAGCCCCAGCCTTCTCCATG-3′) and GPH-AS (5′-GAAATCCCATCACCATCTTCCAGG-3′) from human *GAPDH* (GenBank accession number NM002046).

## 3. Results and Discussion

### 3.1. Structure of the *SUPT16H* Gene with the Integration of a Transposable Element

**Figure 1:**
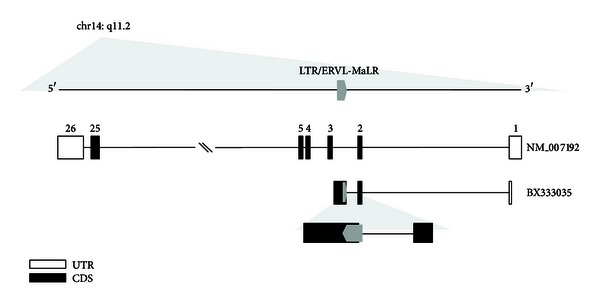
Location and structure of the *SUPT16H* gene. The *SUPT16H* gene is located on chromosome 14q11.2 and includes 26 exons. One of transposable elements (TEs) distributed in genic region of the *SUPT16H* gene, the mammalian apparent LTR retrotransposon (MaLR), is integrated in an intronic region between exon 2 and exon 3. Discriminatively, EST (BX333035) has an alternative exonized MaLR. The structure in the bottom is enlarged exon of BX333035 to show exonized MaLR clearly. UTR: untranslated region; CDS: coding sequence.

The *SUPT16H* gene located on chromosome 14q11.2 encodes accessory factors that may facilitate access to DNA by unpackaging the chromatin structure. It contains 26 exons with several transposable elements such as MaLRs (LTR), Alus (SINE), and L1s (LINE) distributed in the intronic regions. The *SUPT16H* transcript related to the MaLR (MaLR-derived transcript) has 3 exons, and its last exon was created by the integration of the MaLR LTR element in the opposite direction of the host gene (Figure 1).

**Figure 2:**
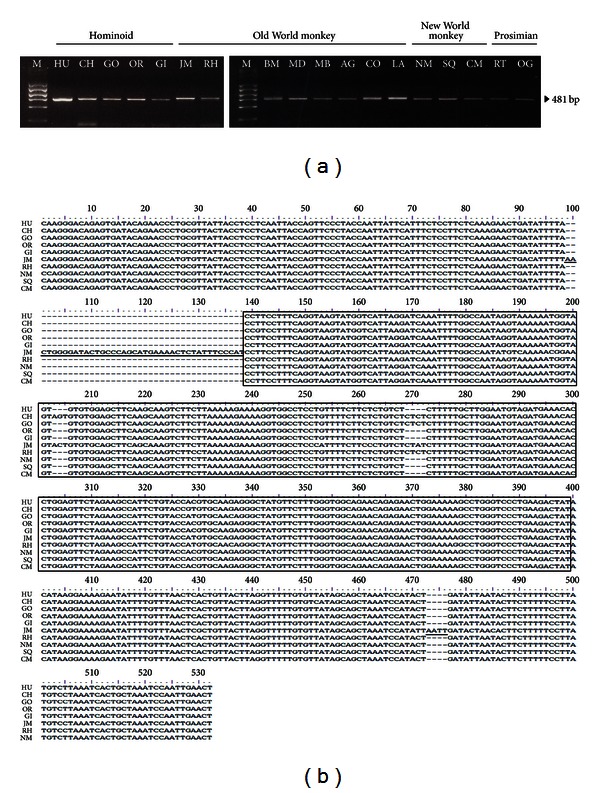
Phylogenetic extent and orthologous structure of the MaLR insertion locus in the primate lineage. (a) Gel chromatographs showing results of the PCR amplification of the MaLR-containing portion of an intron of *SUPT16H* in humans and 17 primates. Species are abbreviated as follows: HU: human; CH: chimpanzee; GO: gorilla; OR: orangutan; GI: gibbon; JM: Japanese macaque; RH: rhesus macaque; BM: bonnet macaque; MD: mandrill; MB: mangabey; AG: African green monkey; CO: colobus; LA: langur; NM: night monkey; SQ: squirrel monkey; CM: common marmoset; RT: ring-tailed lemur; and OG: galago. M indicates a size marker. (b) The alignment of MaLR-containing sequences in primates. The black box and lines indicate MaLR sequences and Japanese-macaque-specific 40 bp and 4 bp sequences, respectively.

MaLRs are considered to function in the mammalian genome by providing alternative splice sites, promoters, and other *cis*-regulatory elements, similar to other TEs that have been conserved during primate evolution [6]. For example, *RNF19* has an alternative transcript resulting from the exonization of MaLR and AluJo. Specifically, the MaLR in the first exon has promoter activity in the sense orientation in the African green monkey fibroblast cell line, Cos7, and the human colorectal carcinoma cell line, HCT116 [22]. As described above, MaLRs are known to be present in genomes of rodents to primates and humans, indicating that they were distributed before the divergence of eutherian mammals. In order to compare the integration time of the MaLR in the *SUPT16H* gene with that from previous reports, we conducted PCR amplification using the genomic DNA of human and other primates (Figure 2). We identified an amplicon that was 481 bp long in all of the primates that we assessed, indicating that the MaLR in the *SUPT16H *gene integrated into the genome of a common ancestor before the divergence of the New World monkey and prosimians. In addition, from the sequencing alignment, we also identified 40 bp and 4 bp of specific sequences in Japanese macaque (Figure 2(b)). These sequences were not detected in any other primate, including the rhesus macaque, which belongs to the Old World monkey family. Thus, these distinct sequences distinguish the Japanese macaque from other primates.

### 3.2. Expressional Analysis of RefSeq mRNA and EST BX333035 of the *SUPT16H* Gene

TEs distributed in the mammalian genome have played important roles in gene regulation and evolution [23]. One of the typical characteristics of TEs which is that they affect the transcription of cellular genes by exonization, which creates an alternatively spliced exon by integration into exonic or intronic regions, has been reported in many studies [24, 25]. Actually, these alternative transcripts are transient and flexible according to the cellular environment and stimuli. However, alternatively spliced variants can be stabilized and increased without selection, and exonization can lead to not only transcriptome diversity but also proteome diversity [2, 10]. Furthermore, these events occur differently or specifically according to the tissue, developmental stage, and disease; thus, alternative transcripts are used to mine disease diagnostic markers [9, 26].

**Figure 3:**
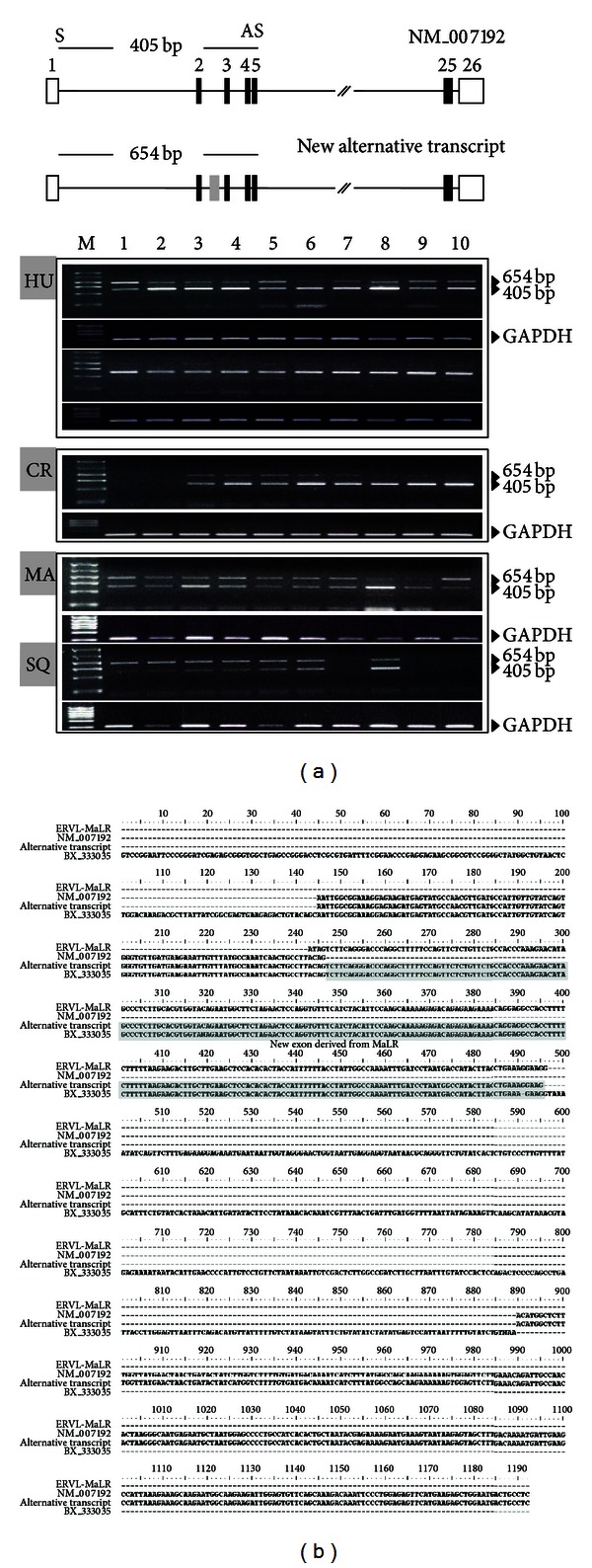
RT-PCR analysis of the *SUPT16H* transcript in human and primate tissues. (a) The *SUPT16H* transcript is expressed broadly in all of the tissues assayed in humans, crab-eating monkey, marmoset, and squirrel monkey. The grey line indicates the position of the primer pair (S: forward primer; AS: reverse primer), and the expected product size is 405 bp. In this analysis, a new alternative transcript was detected that was 654 bp long. It was also expressed broadly. Tissues assayed in humans: M: marker; upper Lane 1: adrenal gland; Lane 2: cerebellum; Lane 3: adult whole brain; Lane 4: heart; Lane 5: kidney; Lane 6: liver; Lane 7: lung; Lane 8: testis; Lane 9: trachea; Lane 10: bone marrow; lower Lane 1: fetal brain; Lane 2: fetal liver; Lane 3: placenta; Lane 4: prostate; Lane 5: salivary gland; Lane 6: skeletal muscle; Lane 7: spinal cord; Lane 8: thymus; Lane 9: thyroid; and Lane 10: uterus. Tissues assayed in crab-eating monkey: Lane 1: cerebrum; Lane 2: cerebellum; Lane 3: pituitary gland; Lane 4: heart; Lane 5: lung; Lane 6: spleen; Lane 7: liver; Lane 8: pancreas; Lane 9: kidney; and Lane 10: urinary bladder. Tissues assayed in marmoset: Lane 1: heart; Lane 2: lung; Lane 3: brain; Lane 4: stomach; Lane 5: liver; Lane 6: kidney; Lane 7: pancreas; Lane 8: large intestine; Lane 9: spleen; and Lane 10: small intestine. Tissues assayed in squirrel monkey: Lane 1: heart; Lane 2: esophagus; Lane 3: interbrain; Lane 4: small intestine; Lane 5: lung; Lane 6: pancreas; Lane 7: cerebrum; Lane 8: large intestine; Lane 9: stomach; and Lane 10: interbrain. *GAPDH* was amplified as a positive control in all instances. (b) The alignment of the *SUPT16H* transcript, new alternative transcript, and EST (BX333035). The alternative transcript has a cassette exon derived from the MaLR (grey box).

To confirm whether the MaLR sequences are expressed and could regulate transcription, we conducted RT-PCR analysis of the RefSeq mRNA (original transcript) and MaLR-derived transcript (BX333035) from several human tissues. Firstly, original transcript was expressed broadly in human tissues. It did not show strong expression or specific expression in particular tissues. We analyzed its expression in tissues of other primates, including the crab-eating monkey, common marmoset, and squirrel monkey (Figure 3). Like the expression in humans, it was ubiquitously expressed in the tissues of these primates. Because we identified japanese monkey-specific sequences, we conducted RT-PCR analysis in rhesus macaque samples to compare the expression in Old World monkeys. However, we could not detect a difference in the *SUPT16H* expression between the japanese monkey and rhesus macaque (data not shown).

**Figure 4:**
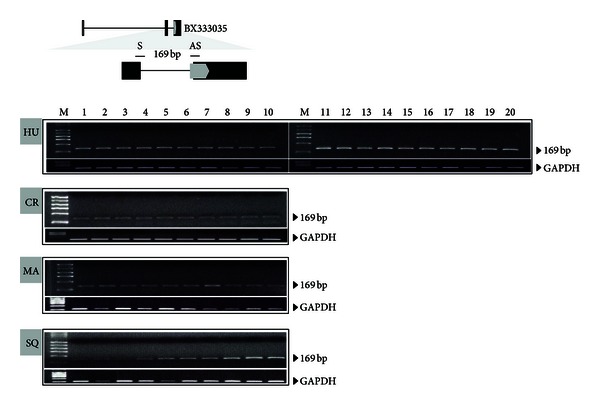
RT-PCR analysis of BX333035 in human and primate tissues. The grey line indicates the position of the primer pair (S: forward primer; AS: reverse primer), and the expected product size is 169 bp. Tissues assayed in humans: M: marker; Lane 1: adrenal gland; Lane 2: cerebellum; Lane 3: adult whole brain; Lane 4: heart; Lane 5: kidney; Lane 6: liver; Lane 7: lung; Lane 8: testis; Lane 9: trachea; Lane 10: bone marrow; Lane 11: fetal brain; Lane 12: fetal liver; Lane 13: placenta; Lane 14: prostate; Lane 15: salivary gland; Lane 16: skeletal muscle; Lane 17: spinal cord; Lane 18: thymus; Lane 19: thyroid; and Lane 20: uterus. Tissues assayed in crab-eating monkey: Lane 1: cerebrum; Lane 2: cerebellum; Lane 3: pituitary gland; Lane 4: heart; Lane 5: lung; Lane 6: spleen; Lane 7: liver; Lane 8: pancreas; Lane 9: kidney; and Lane 10: urinary bladder. Tissues assayed in marmoset: Lane 1: heart; Lane 2: lung; Lane 3: brain; Lane 4: stomach; Lane 5: liver; Lane 6: kidney; Lane 7: pancreas; Lane 8: large intestine; Lane 9: spleen; and Lane 10: small intestine. Tissues assayed in squirrel monkey: Lane 1: heart; Lane 2: esophagus; Lane 3: interbrain; Lane 4: small intestine; Lane 5: lung; Lane 6: pancreas; Lane 7: cerebrum; Lane 8: large intestine; Lane 9: stomach; and Lane 10: interbrain. *GAPDH* was amplified as a positive control in all instances.

Gene expression is a process that produces RNA or proteins through transcription, splicing, and translation. The initiation of gene expression was regulated by chromatin structure. The *SUPT16H* gene is a subunit of the FACT complex that acts as a chromatin-specific transcription elongation factor and interacts with histone H2A/H2B to disassemble nucleosomes and promote transcription elongation [17]. Namely, the broad expression of *SUPT16H* in human and other primate tissues indicated its essential function for gene expression. Next, we analyzed the expression of MaLR-derived transcript. As shown in Figure 4, a transcript related to the MaLR was also expressed broadly in human tissues. This product was also detected in tissues of crab-eating monkey, common marmoset, and squirrel monkey. There was hardly any difference in the expression of 2 transcripts which indicated MaLR did not affect the expression of the *SUPT16H* gene.

### 3.3. Identification of an Alternative Transcript and Its Creation

**Figure 5:**
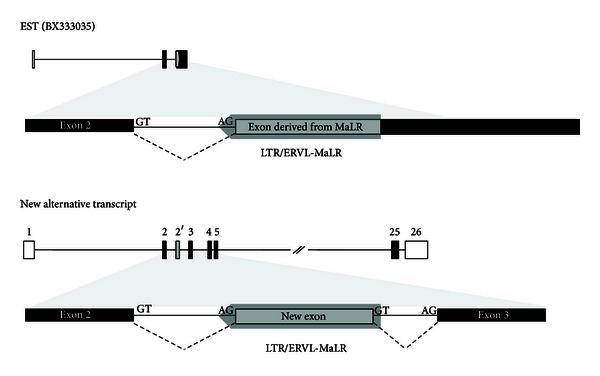
Comparison of structure between MaLR-derived transcript (BX333035) and new alternative transcript. Both MaLR-derived transcript and new alternative transcript have a cassette exon derived from the MaLR in the intron of *SUPT16H*. The MaLR could be exonized by the acceptor site (AG) in its sequence. One difference between the 2 transcripts is the length of the cassette exon. The new alternative transcript has an alternative exon derived from the MaLR only because the MaLR has not only an acceptor site but also a donor site (GT). However, the MaLR-derived transcript has an elongated cassette exon that was formed by combining the new exon and the original exon.

From the RT-PCR analysis, we identified a new alternative transcript. Its product was longer than that of the original transcript because alternative splicing retained an intronic region. This intronic region was derived from the MaLR. The MaLR in the intron was not spliced and exonized (exon 2′) wholly between exons 2 and 3 (Figure 3). Comparing the new alternative transcript and the MaLR-derived transcript (BX_333035), we identified specific features of alternative splicing. Entire sequences of the new exon of the alternative transcript identified in RT-PCR were derived from the MaLR. In addition, this exon was not created by canonical splicing sites. As shown in Figure 5, the MaLR provided both typical alternative splice sites, the acceptor site (AG) and a nucleotide (G) of the donor site (GT). Although these splicing sites were typical sequences recognized by the spliceosome, the splicing pattern could be changed. In case of the transcript related to the MaLR (BX_333035), the MaLR in the intronic region was also exonized to form a new alternative transcript. Strangely, the pattern of splicing was different. Only 1 site, the acceptor site, was recognized by the spliceosome, and the donor site was skipped. Thus, the new exon was elongated. Additionally, the elongated exon included MaLR-derived sequences and sequences of intron and exon 3 of the original and new alternative transcript. In spite of the different splicing pattern, the new alternative transcript was expressed broadly in tissues of humans and other primates, similar to the original and MaLR-related transcripts (Figures 3 and 4).

Alternative splicing is a common mechanism by which most genes are processed [27, 28]. In a previous report, approximately 60% of human genes have at least one alternative transcript [29]. Alternative splicing can be controlled by genetic mutation and epigenetic alteration [30, 31]. Generally, AG and GT are recognized by the spliceosome as the splicing site. However, the spliceosome can recognize splicing sites created by mutation and cryptic splicing sites, leading to alternative splicing [32]. In addition to point mutations, the integration of transposable elements can induce alternative splicing as genetic mutations [2, 25]. If the 2 transcripts showed different and tissue-specific expression patterns, we could assume that tissue-specific factors contributed to alternative splicing; however, both transcripts were expressed broadly in tissues of humans and primates [33]. Alternatively, we could guess that the chromatin structure affected alternative splicing. The mechanism of recognizing splice sites can be altered by epigenetic regulation [34]. Various mechanisms can be considered to understand the differences between the 2 transcripts. Compared to original transcript, the new exon is very unstable and temporary. First, we could expect this new exon was created by cryptic splicing site. However, the new exon includes intronic sequences by the typical splice site, AG-GT (3′–5′), in the new alternative transcript, and AG (3′) in the MaLR-derived transcript existing in the intron. If so, we could guess that the acceptor site and donor site of the new exon could be marked as a weak splice site by epigenetic regulation, such as histone modifications and DNA methylationan, although it was the typical splice site. In a previous study, histone modification was reported as important factor for alternative splicing. Among histone modifications such as H3K36me3, H3K79me1, H2BK5me1, H3K27me1, H3K27me2, and H3K27me3 enriched in exon, the donor site of the new exon could be recognized weakly by the spliceosome with H3K36me3 which is found in weakly expressed exon [35, 36]. Additionally, the DNA methylation and the CG content in the new exonic region could affect the expression of the 2 transcripts. Zhou et al. (2012) reported that exclusive exons have lower levels of CG and methylated CG, whereas retained intron has higher levels [31]. 

## 4. Conclusion

We suggest that the MaLR integrated into an intron of the *SUPT16H* gene before the divergence of New World monkeys and prosimians, and it played a biological role in the evolution of the *SUPT16H* gene during primate evolution by providing an alternative splice site. Although further analyses are required to conclusively elucidate the functions of the transcriptional variants of the *SUPT16H* gene in primate genomes, the results of this study will help us understand the characteristics of the transposable element insertion driving the transcript diversification and its effect on the evolution of the host gene.
